# Evidence on Virtual Reality–Based Therapies for Psychiatric Disorders: Meta-Review of Meta-Analyses

**DOI:** 10.2196/20889

**Published:** 2020-08-19

**Authors:** Laura Dellazizzo, Stéphane Potvin, Mimosa Luigi, Alexandre Dumais

**Affiliations:** 1 Research Center of the Institut Universitaire en Santé Mentale de Montréal Montreal, QC Canada; 2 University of Montreal Montreal, QC Canada; 3 Institut national de psychiatrie légale Philippe-Pinel Montreal, QC Canada

**Keywords:** systematic review, virtual reality, therapy, mental disorders, meta-analysis

## Abstract

**Background:**

Among all diseases globally, mental illnesses are one of the major causes of burden. As many people are resistant to conventional evidence-based treatments, there is an unmet need for the implementation of novel mental health treatments. Efforts to increase the effectiveness and benefits of evidence-based psychotherapy in psychiatry have led to the emergence of virtual reality (VR)–based interventions. These interventions have shown a wide range of advantages over conventional psychotherapies. Currently, VR-based interventions have been developed mainly for anxiety-related disorders; however, they are also used for developmental disorders, severe mental disorders, and neurocognitive disorders.

**Objective:**

This meta-review aims to summarize the current state of evidence on the efficacy of VR-based interventions for various psychiatric disorders by evaluating the quality of evidence provided by meta-analytical studies.

**Methods:**

A systematic search was performed using the following electronic databases: PubMed, PsycINFO, Web of Science, and Google Scholar (any time until February 2020). Meta-analyses were included as long as they quantitatively examined the efficacy of VR-based interventions for symptoms of a psychiatric disorder. To avoid overlap among meta-analyses, for each subanalysis included within this meta-review, only one analysis provided from one meta-analysis was selected based on the best quality of evidence.

**Results:**

The search retrieved 11 eligible meta-analyses. The quality of evidence varied from very low to moderate quality. Several reasons account for the lower quality evidence, such as a limited number of randomized controlled trials, lack of follow-up analysis or control group, and the presence of heterogeneity and publication bias. Nonetheless, evidence has shown that VR-based interventions for anxiety-related disorders display overall medium-to-large effects when compared with inactive controls but no significant difference when compared with standard evidence-based approaches. Preliminary data have highlighted that such effects appear to be sustained in time, and subjects may fare better than active controls. Neurocognitive disorders also appear to improve with VR-based approaches, with small effects being found for various clinical outcomes (eg, cognition, emotion). Finally, there are insufficient data to classify VR-based interventions as an evidence-based practice for social skills training in neurodevelopmental disorders and compliance among patients with schizophrenia.

**Conclusions:**

VR provides unlimited opportunities by tailoring approaches to specific complex problems and individualizing the intervention. However, VR-based interventions have not shown superiority compared with usual evidence-based treatments. Future VR-based interventions should focus on developing innovative approaches for complex and treatment-resistant symptoms that are difficult to address with traditional treatments. Future research should also aim to gain a better understanding of the potential factors that may mediate VR outcomes to improve treatment.

## Introduction

Mental illnesses are one of the predominant causes of burden among all diseases globally [1]. It has been estimated that over 15% of adults in the United States have lived with a psychiatric disorder in the past year, including mental, behavioral, or emotional disorders [2]. Anxiety disorders and depression generally display the highest prevalence rates [2-5]. Furthermore, approximately 4.5% of adults have reported being affected by a severe mental disorder resulting in functional impairment that affects or limits major life activities [2]. In addition, 1 in 6 youths in the United States aged between 6 and 17 years will experience a mental disorder every year [6]. With half of the mental health conditions manifesting by the age of 14 years and three-quarter by mid-20s, youth remains to be an important period for the emergence of a mental disorder [7]. Given the elevated prevalence of mental health problems, psychiatric disorders represent a substantial socioeconomic burden for patients, caregivers, health care providers, and the overall society, with associated costs including informal care, productivity loss, and premature death [1,8,9]. The global direct and indirect economic costs of mental disorders have been estimated at approximately US $2.5 trillion [10]. For those seeking treatment, conventional mental health care for most psychiatric disorders typically includes pharmacological and psychological options. However, there is an enduring discussion as to whether an individual option or a combination of options should be used to treat psychiatric symptoms. Huhn et al [11] conducted a systematic overview of the efficacy of pharmacotherapies and psychotherapies for major psychiatric disorders and concluded that there remains room for amelioration. Among meta-analyses specifically comparing pharmacotherapy with psychotherapy head-to-head, there was a trend in favor of psychotherapy for relapse prevention in depression and bulimia and pharmacological interventions for schizophrenia and dysthymia. Although pharmacological treatments have received more attention, they may likely be less acceptable to patients, and a proportion of individuals will experience adverse effects or will not respond adequately to this approach [12-14]. Evidence-based psychosocial interventions (eg, psychoeducation, interpersonal psychotherapy, cognitive behavioral therapy), offered as the sole or adjunctive treatment, have shown promising results and allow patients to learn skills to overcome and better cope with their symptoms while also preventing relapse [15,16]. Nevertheless, the effect sizes of psychotherapies for mental disorders are moderate at best with dropout rates as high as 30%, and treatment gains not always being maintained for a long term [12,17]. Thus, with underscored inadequacies of conventional treatment, there remains an unmet need for the implementation of novel treatments. Efforts to increase the effectiveness, acceptance, and access to evidence-based psychotherapies have led to the emergence of technology-assisted psychological interventions. A prime example is the virtual reality (VR)–based approach that may enhance conventional face-to-face approaches. Generally, VR techniques are based on similar principles as those used in traditional cognitive behavioral approaches; however, they also increase the possibility of transferring the learning achieved during VR sessions to patients’ everyday lives. These interventions enable the manipulation of the virtual environment and can be used to recreate environmental triggers that elicit distress in patients with mental health problems, thereby allowing them to learn to better manage their difficulties in real time [18,19]. Although VR approaches display additional treatment costs and may lead to cybersickness in some patients [18,20], the literature has nonetheless shown the wide range of advantages of its use, that is, reduced ecological impact, personalized treatment, high level of control over exposure parameters, and better acceptability of and adherence to treatment [18,19,21,22].

VR-based treatments have been developed for many psychopathologies, particularly for anxiety-related disorders, and for developmental disorders, severe mental disorders, and neurocognitive disorders [23,24]. As the field is relatively new, many of these studies have been impacted by methodological issues (ie, small sample size, limited number of randomized trials with strong methodologies including blinding and allocation concealment). Nonetheless, several meta-analyses have been conducted to summarize the evidence of these VR interventions. Statistical meta-analyses are frequently used by clinicians as a resource to determine the best evidence-based treatment options for their patients [25]. Considering the increasing number of meta-analyses on the efficacy of VR-based interventions in psychiatric disorders, we conducted a meta-review to summarize the magnitude of the effects of VR for the treatment of various mental disorders and to evaluate the quality of evidence provided by the meta-analyses. This is to help create recommendations for the use of VR-based approaches for clinicians and policy makers and to guide future research on novel VR interventions.

## Methods

### Search Strategy

A search was independently conducted by 2 graduate students (LD and ML) on PubMed, PsycINFO, Web of Science, and Google Scholar electronic databases, from each database’s inception to February 2020. Search terms were chosen to be inclusive of VR (eg, “virtual,” “virtual reality,” “VR”), mental disorders (eg, “mental illness,” “anxiety,” “post-traumatic stress disorder,” “autism,” “attention deficit hyperactivity disorder,” “neurodevelopmental disorder,” “severe mental disorder,” “depression,” “schizophrenia,” “dementia,” “substance use disorder”), and interventions (eg, “intervention,” “therapy”). The search syntax was tailored for each database. See Multimedia Appendix 1 for the specific search strategy adapted for each database. Only meta-analytical study designs were selected. No setting, date, or geographical restrictions were applied. Searches were limited to English or French language sources. The authors of the articles to which we had restricted access were contacted.

### Study Eligibility

Meta-analyses were included as long as they quantitatively examined the efficacy of VR-based interventions for the symptoms of psychiatric disorders. To maximize the number of meta-analyses, we did not restrict the search to any specific psychiatric population or any age group. It is noteworthy that a problem with meta-analyses is that they may overlap when many have been conducted on a particular disorder and a particular type of subanalysis for the disorder (ie, pre-post efficacy, comparison with inactive or active control, long-term effects). To avoid this issue, for each subanalysis included within this meta-review, only one analysis provided from one meta-analysis was selected based on the best quality of evidence. The inclusion of the meta-analyses was generally based on (1) the year of publication, (2) the number of included studies, and (3) the quality of the included studies (ie, randomized controlled trials [RCTs]). To ensure consensus, discussions on the inclusion of meta-analyses were held with a senior researcher (SP). As a meta-analysis only requires a minimum of 2 studies [26], we chose to include meta-analyses that analyzed at least 2 studies per symptom. However, it should be noted that increasing the number of included studies tends to enhance the generalizability of results [27]. Studies were excluded if they (1) combined several treatment modalities (eg, other computerized approaches such as internet-based therapies) and did not have an effect size for VR specifically or (2) combined disorders together (eg, overall anxiety disorders).

### Data Extraction

Data were extracted using a standardized form by LD and ML. Key information related to the sample, effect sizes (ie, Cohen d, Hedges g, standardized mean difference), outcome measured, control group, timeline (ie, posttreatment, follow-up), confounding factors (ie, moderator analyses), heterogeneity (ie, *Q* statistics, *I^2^* index), and publication bias (ie, funnel plot examination, Egger test) were recorded. Refer to Multimedia Appendix 2 [28-38] for an overview of the extracted data. The effect sizes were categorized as small (0.2), medium (0.5), and large (>0.8) effects [39]. Data were independently extracted by LD and ML, and all queries were resolved in discussions with SP. Furthermore, LD and SP independently undertook quality assessments for the effect sizes reported in the meta-analyses using a set of criteria based on the Grading of Recommendation, Assessment, Development, and Evaluation checklist [40-43]. Higher scores were assigned to analyses that suggested more precision (ie, a smaller range of 95% CIs around the effect size [under 0.5 absolute effect size]), analyzed follow-ups, included only controlled trials, conducted moderator analyses, reported no heterogeneity and publication bias, and included an outcome principally targeted by the intervention. Studies were assigned to be of high, moderate-to-high, moderate, moderate-to-low, low, and very low quality. To achieve a high standard of reporting data, the PRISMA (Preferred Reporting Items for Systematic Reviews and Meta-Analyses) guidelines were followed (Multimedia Appendix 3) [44].

## Results

### Description of Studies

**Figure 1:**
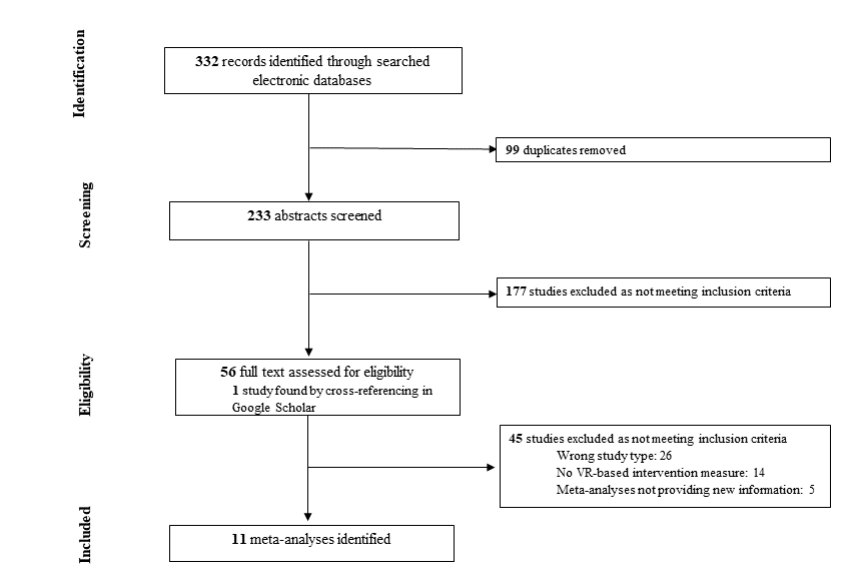
Flow-chart depicting the search strategy employed to find the meta-analyses to include in this review.

The literature search identified 233 potential articles that were screened for eligibility after removing duplicates. One additional meta-analysis was identified by cross-referencing on Google Scholar. Among these articles, 11 meta-analyses were selected that provided 41 effect sizes. The PRISMA flowchart for the inclusion of studies in the meta-review is shown in Figure 1. The psychiatric disorders were categorized based on the Diagnostic and Statistical Manual of Mental Disorders, 5th Edition [45], as (1) anxiety disorders (acrophobia, arachnophobia, aviophobia, panic disorder, and social anxiety), (2) trauma- and stress-related disorders (posttraumatic stress disorder), (3) severe mental disorders (depressive disorder and schizophrenia spectrum), (4) neurodevelopmental disorders (autism), and (5) neurocognitive disorders (mild cognitive impairment and dementia). When several analyses were conducted within the meta-analyses, we retrieved one specific effect size estimate for (1) the pre-post efficacy of VR-based interventions, (2) the comparison of VR-based interventions with inactive control, (3) the comparison of VR-based interventions with active controls, and (4) the long-term effects of VR-based interventions after the follow-up. Refer to Multimedia Appendix 2 [28-38] for a summary of the quality of evidence provided by the included meta-analyses. Each meta-analysis included 2-16 studies, with samples ranging between 30 and 454 individuals.

### Anxiety Disorders

Anxiety disorders, particularly specific phobias (eg, fear of flying, fear of heights), have become typical in VR implementation as exposure is undeniably a key element that must be addressed in these disorders [28,46-48]. There are 2 predominant theoretical models related to learning, which may explain how exposure therapy reduces anxiety [49]: emotional processing theory [50,51] and inhibitory learning model [52]. Both theories claim that exposure allows patients to learn corrective information about a stimulus that is feared. In VR-based interventions, the sense of presence or the feeling of *being there* has been considered as the principle mechanism that leads to the experience of anxiety [53,54]. In this sense, the feeling of presence experienced in VR offers the opportunity to immerse patients to their feared stimuli in the VR environment, which is customized to match specific aspects of their fear [55,56].

#### Specific Phobias

##### Fear of Heights (Acrophobia)

A meta-analysis by Parsons et al [29] found an average random effect size of 0.93 (95% CI 0.44 to 1.43) for acrophobia. Graded as low-quality evidence from 4 studies with different control groups (2 were compared with a waitlist, 1 had no control group, and 1 was compared with in vivo interventions), the results of this meta-analysis suggest a statistically large overall effect for this specific phobia. However, heterogeneity and publication bias were not evaluated, and confounding factors were not considered.

##### Fear of Spiders (Arachnophobia)

A meta-analysis by Parsons et al [29], comprising 4 studies with mixed designs, found an overall large effect of 0.92 (95% CI 0.25 to 1.59) for VR interventions. The evidence was graded as low quality, notably owing to the inclusion of studies with mixed designs and the lack of consideration of heterogeneity, publication bias, and confounding factors. Better quality evidence was provided by Opris et al [28], including RCTs that compared VR-based interventions with active controls specifically. The authors retrieved 2 studies that showed no significant posttreatment (d=−0.12; 95% CI −0.31 to 0.06) and no long-term (d=−0.20; 95% CI −0.49 to 0.08) differences in primary arachnophobia outcomes.

##### Fear of Flights (Aviophobia)

Evidence based on the meta-analysis by Cardoş et al [30] including RCTs for aviophobia was evaluated to be of low-to-moderate to moderate quality. First, regarding the efficacy of VR-based interventions based on a large sample size of 454 participants, 16 study arms were included at posttreatment and 15 at follow-up. Statistically significant medium effect sizes were observed (g=0.592; 95% CI 0.327 to 0.858; g=0.588; 95% CI 0.216 to 0.960, respectively). As both analyses presented statistically significant heterogeneity, moderator analyses were conducted to explain the divergences among studies. The quality of randomized trials and the mean age of patients were significant moderators at posttreatment, whereas the number of patients and follow-up intervals were significant moderators at follow-up. Moreover, examination of the funnel plot showed asymmetry, suggesting publication bias. Second, when compared with the inactive control groups, the results showed large statistically significant effects at posttreatment (g=1.350; 95% CI 0.664 to 2.037) and medium statistically significant effects at follow-up (g=0.583; 95% CI 0.108 to 1.058). Heterogeneity was observed at posttreatment, and there was presence of funnel plot asymmetry. Third, when comparing VR with classical evidence-based interventions, the results showed a small significant effect for VR-based interventions (g=0.353; 95% CI 0.152 to 0.555), whereas follow-up studies indicated a moderate significant effect (g=0.615; 95% CI 0.179 to 1.052). Heterogeneity was evident at follow-up, with the number of participants and follow-up period as significant moderators. Furthermore, follow-up studies pointed toward publication bias. Fourth, a lack of difference between VR-based interventions and other exposure-based interventions was observed at posttreatment (g=0.122; 95% CI −0.225 to 0.469). A moderate-to-large significant effect was still found at follow-up, in favor of VR-based interventions (g=0.697; 95% CI 0.101 to 1.292). Significant heterogeneity was observed, revealing 3 moderators (number of exposure sessions, outcome type, and follow-up intervals), in addition to the presence of publication bias.

In summary, meta-analytical evidence shows that VR-based interventions may be effective for specific phobias. The quality of evidence ranged from low to moderate quality, with better quality evidence provided for aviophobia, which comprised a larger number of RCTs with a larger sample size. The presence of heterogeneity and publication bias was evaluated for aviophobia. When compared with active controls, the results suggested better aviophobia outcomes for VR-based therapies than classical evidence-based interventions, with no significant superiority over other exposure-based therapies. However, for arachnophobia, significant superiority was found for VR when compared with active controls. Finally, the effects of VR for aviophobia remained stable in time, indicating that VR might fare better than active controls in the long term.

#### Panic Disorder With or Without Agoraphobia

First, the meta-analysis by Parsons et al [29] included 3 studies and observed very large significant overall effects (d=1.79) for VR-based interventions at posttreatment. The evidence was graded to be of low quality, notably owing to the inclusion of studies with mixed designs and the lack of consideration for heterogeneity, publication bias, and confounding factors. Second, Fodor et al [31] observed large effects at posttreatment for VR-based interventions in comparison with inactive controls (g=1.80; 95% CI 1.01 to 2.60). The evidence provided by 2 RCTs was graded as low-to-moderate quality; the authors did not observe any heterogeneity, although there was a publication bias for their entire study sample. Third, Fodor et al [31] found no significant difference at posttreatment between VR and other psychological therapies (g=−0.05; 95% CI −0.32 to 0.21). This evidence graded as moderate quality was provided from 6 RCTs, and the data displayed no heterogeneity. Finally, Opris et al [28] analyzed the long-term effect of VR-based therapies, specifically in comparison with active controls. Their analysis found a small significant effect favoring VR (d=0.18; 95% CI 0.10 to 0.26). Evidence was graded as low-to-moderate quality based on 2 RCTs. However, heterogeneity and publication bias were not reported.

In summary, these meta-analyses on panic disorder with or without agoraphobia showed that VR-based interventions are efficient. Evidence has been evaluated to be of low to moderate quality, with the quality of evidence being lower owing to a lack of consideration of heterogeneity, publication bias, and moderating factors. Better quality evidence was provided for comparison with classical evidence-based interventions, which showed that VR was no better than these interventions at posttreatment. At follow-up, there was a small superiority observed favoring VR over standard interventions.

#### Social Anxiety

First, the meta-analysis by Kampmann et al [32] observed large overall effects for VR-based interventions at posttreatment (g=1.09; 95% CI 0.80 to 1.39) of social anxiety symptoms. Evidence provided by 3 RCTs was graded as low-to-moderate quality, with heterogeneity, publication bias, and moderators not being examined. Second, in comparison with inactive controls, the meta-analysis by Carl et al [33] found a large posttreatment effect (g=0.97; 95% CI 0.62 to 1.31). Evidence from 7 studies included randomized controls, with a total sample of 236 individuals, and was evaluated to be of low-to-moderate quality. Although not specifically for this subanalysis, the authors did observe moderate heterogeneity and possible presence of publication bias in their overall study. Third, as for the comparison with active controls, Chesham et al [34] found no significant difference between VR-based interventions and standard treatments using in vivo or imaginal approaches (g=−0.01; 95% CI −0.30 to 0.28). Evidence from 7 well-controlled trials (n=340) with moderate heterogeneity and no presence of publication bias was graded as low-to-moderate quality. Fourth, in terms of follow-up assessments, Kampmann et al [32] observed that the large overall effect for VR was maintained in time (g=0.93 for less than 5 months and g=1.20 for over 5 months). However, the effect was not different from the effect of active controls. Evidence was evaluated as low-to-moderate quality as the authors did not examine heterogeneity or publication bias owing to the limited number of trials included in their analyses.

In summary, overall evidence was evaluated as low-to-moderate quality: most meta-analyses included a limited number of trials and moderator analyses, and did not report heterogeneity or publication bias. Medium to large effects were observed for VR-based interventions for social phobia. Nevertheless, no significant difference existed between the VR-based interventions and standard treatment. The overall beneficial effect of VR interventions was maintained in the long term, although no significant difference was observed with active controls.

### Trauma- and Stressor-Related Disorders

Trauma- and stress-related disorders (such as posttraumatic stress disorder) may develop by directly experiencing, witnessing, or repeating exposure to aversive elements of a traumatic event (eg, combat, sexual assault). Although many show resilience following exposure, up to one-third of those confronted with a traumatic event will subsequently develop clinically relevant posttraumatic symptoms (eg, reexperiencing, avoidance) [57]. It is worth noting that VR exposure therapy has potential efficacy in the treatment of posttraumatic stress disorder for different types of trauma and that this technology can compensate for the shortcomings of traditional therapy (ie, inherent avoidance of traumatic memory) [58,59]. VR may ease the emotional engagement of patients during exposure to traumatic stimuli by eschewing avoidance symptoms and facilitating therapeutic control [60].

First, a meta-analysis by Deng et al [35] found a small superiority of VR interventions for posttraumatic stress disorder symptoms in comparison with inactive and active controls combined. This effect was significant (g=0.327; 95% CI 0.105 to 0.550). A similar significant effect of VR-based interventions was observed when considering only studies that used intention-to-treat analyses or reported complete outcome data (g=0.584; 95% CI 0.318 to 0.850). Evidence was evaluated to be of moderate quality, provided from 10 RCTs (n=309) showing moderate heterogeneity and no publication bias. Second, the same authors observed moderate effects for the superiority of VR-based interventions relative to inactive controls alone (g=0.567; 95% CI 0.270 to 0.863) [35]. Evidence was evaluated to be of low-to-moderate quality based on 5 RCTs (n=175) with no publication bias; heterogeneity for this specific subanalysis was not provided. Third, no significant difference was found between VR and active controls [35]. Evidence from 6 RCTs (n=239) was also evaluated to be of low-to-moderate quality with no presence of publication bias; heterogeneity for this specific subanalysis was similarly not provided. Finally, as for follow-up effects, Deng et al [35] found moderate-to-large improvements for VR-based interventions in comparison with the combination of inactive and active controls (g=0.697 and g=0.848 for short- and long-term effects, respectively). Evidence provided by 9 and 11 RCTs was evaluated to be of low-to-moderate quality. Moderator analyses, heterogeneity, and publication bias were not reported.

In summary, the meta-analyses on posttraumatic stress disorder showed small-to-moderate effects for VR. Evidence was generally graded as low-to-moderate to moderate quality. No significant difference was found with standard evidence-based interventions. Moreover, improvements in VR were maintained in time.

### Severe Mental Disorders

VR-based treatments for the symptoms of individuals with severe mental disorders have multiplied in recent years. Although there are very limited studies on the effects of VR for those with mood disorders [61,62], this innovative tool may nonetheless be used to deliver psychoeducation and to induce relaxation and enhance positive emotions [63]. Moreover, VR scenarios have been used to treat symptoms of other severe mental disorders such as schizophrenia by enabling patients to practice social skills (eg, vocational skill training) and learn to cope with distress associated with psychotic symptoms [64,65]. As those with severe mental disorders also experience difficulties with activities in everyday life, VR may also be used to test and support their performance using an environment that simulates real-life activities and increase compliance with treatment [36].

#### Depressive Disorder

It is worth noting that VR interventions included in the meta-analyses did not target depression as a diagnosis per se and did not generally aim at the reduction of depressive symptoms as a main outcome. First, an overall small effect was observed for VR-based interventions at posttreatment in the analysis by Kampmann et al [32] (g=0.44; 95% CI 0.02 to 0.87). Evidence evaluated to be of low-to-moderate quality was based on 2 randomized trials (n=119); moderator analyses, heterogeneity, and publication bias were not reported. Second, when compared with inactive controls, Fodor et al [31] found a significant moderate effect for VR interventions (g=0.73; 95% CI 0.25 to 1.21). Evidence graded as low-to-moderate quality was based on 10 RCTs showing high heterogeneity; there was also the presence of publication bias on their overall analyses. Third, the same authors observed no significant difference between the VR and active controls at posttreatment. Evidence was evaluated as moderate quality based on 13 RCTs showing low heterogeneity. Fourth, in the follow-up assessment by Fodor et al [31], which retrieved 5 RCTs, there was no significant difference in comparison with active controls. Moderate heterogeneity was observed, and evidence was similarly graded as moderate quality.

In summary, evidence from these meta-analyses on depressive symptoms highlighted that overall VR-based interventions may reduce comorbid depressive symptoms. Evidence was graded as low-to-moderate quality. However, the effect did not seem to be different from standard evidence-based interventions. No significant long-term differences were found at follow-up compared with active controls.

#### Schizophrenia Spectrum Disorders

A meta-analysis by Valimaki et al [36] investigated RCTs on the effects of VR to support treatment compliance among patients with schizophrenia spectrum disorders. Treatment compliance was defined as loss to follow-up and withdrawal by the trialist. Overall, 3 short-term trials (n=156) with a duration of 5 to 12 weeks were retrieved, which were aimed at delivering skill training (ie, social skills and vocational skills). The authors assessed the quality of the included trials as low quality. Findings showed that there was a nonsignificant effect of VR on compliance (risk difference=0.02; 95% CI −0.08 to 0.12). Evidence provided by this meta-analysis was evaluated to be of moderate quality, showing no heterogeneity yet a moderate risk of bias. Comparison with active controls has not been reported.

In summary, at present, there are insufficient quality data to classify VR as an evidence-based practice for treatment compliance in patients with schizophrenia.

### Neurodevelopmental Disorders

Autism spectrum disorder has received interest in the field of VR. VR technologies have been promising by supporting learning for children and adults with autism, who may find social interactions difficult. Several VR environments have been developed, such as virtual cafes, schools, or job interviews [66]. VR allows role play and practice skills without the threat of real-world consequences [67].

One meta-analysis by Barton et al [37] evaluated the effects of technology-aided support in comparison with a control condition on improving a mix of primary skills (ie, communication, academic, engagement or task completion, social, emotion recognition, and adaptive). For VR-based interventions specifically, 2 studies using group designs amounting to a small sample size of 30 individuals were included. One study comprised children with high-functioning autism for social interaction training and the other study comprised adults with autism spectrum for job interview training. Evidence was thus evaluated to be of very low quality, with heterogeneity and publication bias not being reported for the subanalysis. The meta-analysis yielded a nonsignificant estimated effect size of 0.37 (95% CI −1.71 to 2.46). Moreover, follow-up results were not evaluated.

In summary, there are insufficient quality data to classify VR as an evidence-based practice among individuals with autism.

### Neurocognitive Disorders

Individuals with neurocognitive disorders (ie, mild cognitive impairment or dementia) may benefit from VR-based interventions that promote simulations of functional learning, the transfer of learned functions to daily life, and relaxation [38].

A meta-analysis by Kim et al [38] analyzed the effects of different VR-based intervention platforms for individuals with mild cognitive impairment and dementia. The authors found an overall small effect size for VR, including executive, emotion, fitness, and cognitive outcomes (d=0.29; 95% CI 0.16 to 0.42). Larger improvements were found for patients with mild cognitive impairment compared with patients with dementia or mixed samples. With regard to their subanalysis for experimental and control group allocation, random allocation (d=0.36) and no randomization (d=0.4) showed small-to-moderate effects, which were larger than those with a one-group design (d=0.15). When subdividing the different outcomes, the effect sizes for cognitive functions (d=0.42) were higher and significant in comparison with emotion (d=0.14) and executive functions (d=0.07). Overall, evidence graded as low to low-to-moderate quality was based on a mix of impairments provided from 11 studies with mixed designs with significant heterogeneity; the authors stated that publication bias was not a concern. There was also a lack of follow-up assessments and no comparison with active controls.

In summary, low to low-to-moderate quality evidence indicated that VR interventions may positively affect various clinical outcomes among patients with cognitive impairment and thus improve cognitive and routine functions. However, these VR-based interventions were not compared with active controls.

## Discussion

This meta-review aimed to summarize the current state of evidence on the efficacy of VR-based interventions for psychiatric disorders by evaluating the data provided by meta-analytical studies. Cumulating evidence on various anxiety disorders and posttraumatic stress disorder showed that VR-based interventions displayed overall medium-to-large effects in comparison with inactive controls. However, there was globally no significant difference in comparison with standard evidence-based approaches at posttreatment, apart from significant differences with classical evidence-based interventions (g=0.353 in favor of VR) for aviophobia. With limited evidence on the superiority of one over the other, these findings suggest that both VR-based and standard evidence-based therapies are as effective for anxiety-related disorders at posttreatment. Furthermore, although results may not be dissimilar among interventions in the short term, preliminary data on aviophobia and panic disorder have highlighted that the effects of VR appear to be sustained in time, and subjects may fare better in the long term than with active controls. This suggests that although the effects of conventional treatments diminish in time, the effects of VR appear to be maintained, leading to longer-lasting positive outcomes. Such differential outcomes may be explained by the advantages of VR over classical and in vivo exposure-based interventions, comprising a more flexible and personalized approach where the therapist can better control the content of exposure (eg, including turbulence in the exposure of flight phobia), exposure rhythm, and repetition of scenarios [68-70].

VR interventions have also shown promise in the treatment of other disorders included in this meta-review. First, although there are only a few VR interventions that have been developed specifically for individuals with mood disorders [61,62], VR-based therapies have been reported to be effective in the short term to reduce depressive symptoms comorbid with anxiety-related disorders. Second, neurocognitive disorders seem to benefit from VR-based interventions with overall small effects on clinical outcomes such as cognition and emotion. The interaction provided in VR environments may therefore improve well-being, routine functions, and cognition among patients with cognitive impairments by stimulating them. However, it is unknown if VR fares better than conventional treatments for this population. As for autism, given the core impairments in social communication and interaction, it is contended that VR may have a potential for training in highly controlled social scenarios, allowing patients to rehearse interactions or responses [66]. However, no support was observed for the efficacy of VR for neurodevelopmental disorders on social skills training, but this was based on limited low-quality studies on autism [66]. Similarly, VR environments have been created to enable skills training of everyday tasks among patients with schizophrenia, such as to help support treatment follow-up and medication taking. However, the retrieved meta-analysis for compliance among patients with schizophrenia yielded no significant effects based on the low-quality studies included [36].

As observed, meta-analyses serve as a useful tool to provide a global overview of the benefits of VR for patients affected by psychiatric disorders. The quality of evidence was evaluated as being quite variable, ranging from very low to moderate quality. Several reasons account for the lower quality of evidence. First, many meta-analyses included a limited number of RCTs within their analyses, thereby also lacking large sample sizes. Meta-analyses with a larger number of randomized trials were provided for aviophobia [30], posttraumatic stress disorder [35], and depressive symptoms [31]. Among these meta-analyses, Cardoş et al [30] conducted moderator analyses and observed that the quality of RCTs was a significant moderator, with lower quality trials yielding larger effect sizes. Although few highlighted this as a concern, it remains of importance because an RCT with methodological issues, such as lack of blinding, is insufficient to create evidence-based practice. Thus, the quality of the studies should be taken into account to understand the efficacy of interventions. Second, for certain psychiatric disorders such as autism and mild cognitive impairment and dementia, no analysis was conducted evaluating long-term effects and comparing VR-based interventions with active controls. Moreover, the outcomes were less well-defined in both disorders. Third, when they were reported, results often displayed moderate-to-high heterogeneity, which may suggest the presence of subgroups of patients that may better respond to VR than others. Unfortunately, most meta-analyses did not report heterogeneity. Finally, although several meta-analyses did not report publication bias, many noted the presence of publication bias, which may suggest the possibility of either overestimating or underestimating the results. This reinforces the importance of registering the conducted studies.

Furthermore, numerous psychiatric symptoms and disorders that are treatable by VR interventions have not been examined by meta-analytical investigation. For example, although we retrieved only one meta-analysis on compliance for schizophrenia [36], the past decade has seen an emergence in VR treatment for other symptoms such as positive symptoms of psychosis (ie, delusions and auditory verbal hallucinations). These therapies have shown important benefits in psychotic symptomatology, with large effects being observed for both delusions and auditory verbal hallucinations in several trials [71-74]. As a second area of interest, traditional psychological interventions in the field of addiction generally teach individuals new skills to avoid high-risk situations, to refuse substance offers, and to ultimately better cope with cue- and stress-related craving. However, these conventional treatments, such as imaginal cue exposure therapy, have provided mixed findings [75-78], which may be improved by using VR [79]. VR technology may add effectiveness to standard treatments (ie, cue exposure treatment) owing to its capacity to induce greater subjective and physiological craving, which may prompt the generalization of treatment effects to real-life daily activities [80]. A limited amount of research has been conducted to date on the efficacy of VR-based cue exposure approaches for addiction, which aim to extinguish craving and prevent relapses. Promising results from case reports and small trials on subjective and physiological outcomes have emerged for nicotine [81-83], alcohol use disorder [80,84,85], and pathological gambling [86]. Furthermore, research on treatments for eating disorders has paralleled the methods used in the treatment of addiction and adapted them for food cues and environmental settings key to eating behaviors [87]. These interventions have aimed to improve eating disorders, with outcomes including craving, weight regain, and eating patterns [88-91]. Finally, VR may show potential for the treatment of more deviant behaviors such as violence-related outcomes in psychiatric samples. VR may provide a solution to the shortcomings of conventional interventions for violence (ie, clinicians cannot ethically place offenders in at-risk situations) by enabling individuals to be immersed into virtual simulations of real-life events under the control of the clinician [92,93]. Preliminary studies in at-risk populations have shown reductions in anger and impulsivity, improvements in conflict resolution skills and empathy levels, and decreases in aggression [94]. Hitherto, clinical research with novel VR development could make an important contribution to patient care [64], mostly when traditional face-to-face interventions may be more limited or cannot be conducted. Although no meta-analytical evidence was available for the disorders stated above at the time of our literature search, there is preliminary support for the use of VR-based interventions to improve the treatment of symptoms of other psychiatric disorders. Nevertheless, research remains to be generally limited by fewer studies, small samples, lack of control groups (mainly standard evidence-based interventions), and lack of follow-up. In this sense, future research using strong methodology (ie, single-blinded RCTs with large samples) is required to determine whether VR approaches yield additional benefits over standard treatment and whether these effects last over time.

In the above efficacy studies, some key aspects remain to be further investigated. With the rise of personalized medicine, future research should be encouraged to achieve a better understanding of factors that may play a role in VR outcomes and help explain different effects from usual treatment. These factors may include patient characteristics (eg, age, gender, and personality traits) and the severity of the disorder (eg, comorbidities, treatment resistance); certain patients may indeed be more susceptible to better respond to these VR approaches. For instance, a meta-analysis by Cardoş et al [30] on the symptoms of aviophobia found that the age of the participants was a significant moderator, explaining the difference in efficacy of VR-based interventions at posttreatment, with greater effects among younger individuals. In addition, the design of the virtual environments and exposure approach of the therapy may have a role in the therapeutic outcome, which warrants further investigation. Hence, it may be suggested that patients who fully experience the VR paradigm as realistic (ie, higher level of immersion and sense of presence) may respond better to the intervention [31,64]. This may be possible with the use of more recent technologies, which are more immersive and closely resemble the real world. Improved engagement with the virtual environment, with the inclusion of social dynamic interactions via tailored avatars, may similarly have a role in the efficacy of the intervention and heighten the sense of presence and immersion [79]. These dynamic interactions may enable patients to engage with the VR environment in a more naturalistic and intuitive way [95,96]. It is noteworthy that the sense of immersion may be increased by incorporating senses other than vision into the VR environment, such as hearing and smell. Supplemental studies are needed to evaluate the effects of these factors to possibly improve the efficacy of VR-based treatments.

### Conclusions

VR provides opportunities to go over and beyond traditional interventions and allows tailoring approaches to each individual, thereby possibly improving efficacy and the maintenance of skills. With variable quality of evidence, meta-analytical literature suggests positive outcomes in the VR treatment of psychiatric conditions, mainly anxiety-related disorders. VR-based interventions are better than inactive controls and generally show similar effects when compared with evidence-based approaches for these disorders. Preliminary findings also suggest that the effects of VR may be long-lasting. Furthermore, VR has shown efficacy for the treatment of depressive symptoms and neurocognitive disorders. However, support for the use of VR in the treatment of social skills in autism and compliance in schizophrenia is lacking. There are also numerous VR studies that were not included in meta-analyses that targeted other psychiatric symptoms and disorders (ie, psychotic symptoms, addiction); these have also shown prefatory beneficial outcomes. Nevertheless, more research is necessary in the field of psychiatry to establish high-quality evidence with the use of gold-standard evidence from well-designed RCTs comprising large samples. As current VR treatments have not clearly shown superiority over conventional treatments, future VR-based interventions should focus on developing innovative approaches for complex and treatment-resistant symptoms that are difficult to address with traditional treatment. Research is also warranted to evaluate the aspects enabling the better use of VR and examine the specificity of VR-based interventions. As soon as more studies become available, systematic meta-regression analyses could statistically examine the influence of certain variables on the efficacy of VR for improving personalized patient care.

## Appendix

Multimedia Appendix 1 Electronic search strategy for the meta-review conducted.

Multimedia Appendix 2 Details of the retrieved studies included in the meta-review.

Multimedia Appendix 3 PRISMA (Preferred Reporting Items for Systematic Reviews and Meta-Analyses) Checklist.

## Abbreviations

PRISMA: Preferred Reporting Items for Systematic Reviews and Meta-Analyses
RCT: randomized controlled trial
VR: virtual reality
