# Supplementary material for: Evidence on Virtual Reality–Based Therapies for Psychiatric Disorders: Meta-Review of Meta-Analyses
Source: J Med Internet Res. 2020 Aug 19;22(8):e20889. doi: 10.2196/20889 (PMC7468638; doi:10.2196/20889)
Supplement: Multimedia Appendix 1 [file jmir_v22i8e20889_app1.docx]

**Supplementary Online Content**

Dellazizzo, L., Potvin, S., Luigi, M. & Dumais A. Evidence on virtual reality-based therapies for psychiatric disorders: Meta-review of meta-analyses

**Multimedia Appendix 1.** Electronic search strategy for the meta-review conducted.

This supplementary material has been provided by the authors to give readers additional information about their work.

**Multimedia Appendix 1. Electronic search strategy for the meta-review conducted.**

| **Database; Search** | **Search terms** |
| --- | --- |
| 1. **PubMed**;   k= 96 | ("meta-analysis"[Title/Abstract] OR "meta-regression”[Title/Abstract] OR “meta-analytic*”[Title/Abstract] OR “systematic review”[Title/Abstract]) AND (“virtual reality”[Title/Abstract] OR “VR”[Title/Abstract] OR “virtual”[Title/Abstract] OR “computer-assisted”[Title/Abstract]) AND ("treatment"[Title/Abstract] OR "therapy"[Title/Abstract] OR "intervention"[Title/Abstract] OR "virtual reality exposure therapy"[Title/Abstract] OR “psychotherapy”[Title/Abstract]) AND (“anxiety”[Title/Abstract] OR “anxiety disorder”[Title/Abstract] OR “depression”[Title/Abstract] OR “depressive disorder”[Title/Abstract] OR “mood disorder”[Title/Abstract] OR “schizophrenia”[Title/Abstract] OR “psychosis”[Title/Abstract] OR “psychotic”[Title/Abstract] OR “phobia”[Title/Abstract] OR “PTSD”[Title/Abstract] OR “Post-traumatic stress disorder”[Title/Abstract] OR “substance use disorder”[Title/Abstract] OR “substance abuse”[Title/Abstract] OR “addiction”[Title/Abstract] OR “alcohol use disorder”[Title/Abstract] OR “autism”[Title/Abstract] OR “anorexia”[Title/Abstract] OR “bulimia”[Title/Abstract] OR “eating disorder”[Title/Abstract] OR “binge eating”[Title/Abstract] OR “obsessive-compulsive disorder”[Title/Abstract] OR “ADHD”[Title/Abstract] OR “attention deficit hyperactive disorder”[Title/Abstract] OR “dementia”[Title/Abstract] OR “cognitive impairment”[Title/Abstract] OR “psychiatric”[Title/Abstract] OR “mental illness”[Title/Abstract] OR “mental health”[Title/Abstract]) |
| 1. **PsycINFO**;   k= 55 | ( (Abstract:("meta-analysis")) OR (Abstract:(“meta-regression”)) OR (Abstract:(“meta-analytic”)) OR (Abstract:("systematic review")) OR (Title:("meta-analysis")) OR (Title:(“meta-regression”)) OR (Title:(“meta-analytic”)) OR (Title:("systematic review")) ) AND ( (Abstract:("virtual reality")) OR (Abstract:(“VR”)) OR (Abstract:(“virtual”)) OR (Abstract:("computer-assisted")) OR (Title:("virtual reality")) OR (Title:(“VR”)) OR (Title:(“virtual”)) OR (Title:("computer-assisted")) ) AND ( (Abstract:("treatment")) OR (Abstract:(“therapy”)) OR (Abstract:(“intervention”)) OR (Abstract:("virtual reality exposure therapy")) OR (Abstract:("psychotherapy")) OR (Title:("treatment")) OR (Title:(“therapy”)) OR (Title:(“intervention”)) OR (Title:("virtual reality exposure therapy")) OR (Title:("psychotherapy")) ) AND ( (Abstract:("anxiety")) OR (Abstract:(“anxiety disorder”)) OR (Abstract:(“depression”)) OR (Abstract:("depressive disorder")) OR (Abstract:("mood disorder")) OR (Abstract:("schizophrenia")) OR (Abstract:(“psychosis”)) OR (Abstract:(“psychotic”)) OR (Abstract:("phobia")) OR (Abstract:("PTSD")) OR (Abstract:("post-traumatic stress disorder")) OR (Abstract:(“substance use disorder”)) OR (Abstract:(“substance abuse”)) OR (Abstract:("addiction")) OR (Abstract:("alcohol use disorder")) OR (Abstract:("ADHD")) OR (Abstract:(“attention deficit hyperactive disorder”)) OR (Abstract:(“autism”)) OR (Abstract:("anorexia")) OR (Abstract:("bulimia")) OR (Abstract:(“eating disorder”)) OR (Abstract:(“binge eating”)) OR (Abstract:("obsessive-compulsive disorder")) OR (Abstract:("dementia")) OR (Abstract:("cognitive impairment")) OR (Abstract:(“psychiatric”)) OR (Abstract:(“mental illness”)) OR (Abstract:("mental health")) OR (Title:("anxiety")) OR (Title:(“anxiety disorder”)) OR (Title:(“depression”)) OR (Title:("depressive disorder")) OR (Title:("mood disorder")) OR (Title:("schizophrenia")) OR (Title:(“psychosis”)) OR (Title:(“psychotic”)) OR (Title:("phobia")) OR (Title:("PTSD")) OR (Title:("post-traumatic stress disorder")) OR (Title:(“substance use disorder”)) OR (Title:(“substance abuse”)) OR (Title:("addiction")) OR (Title:("alcohol use disorder")) OR (Title:("ADHD")) OR (Title:(“attention deficit hyperactive disorder”)) OR (Title:(“autism”)) OR (Title:("anorexia")) OR (Title:("bulimia")) OR (Title:(“eating disorder”)) OR (Title:(“binge eating”)) OR (Title:("obsessive-compulsive disorder")) OR (Title:("dementia")) OR (Title:("cognitive impairment")) OR (Title:(“psychiatric”)) OR (Title:(“mental illness”)) OR (Title:("mental health")) ) |
| 1. **Web of Science**;   k= 181 | (TS=(meta-analysis OR meta-regression OR meta-analytic OR systematic review) AND TS=(virtual reality OR VR OR virtual OR computer-assisted) AND TS=(treatment OR therapy OR intervention OR virtual reality exposure OR psychotherapy) AND TS=(anxiety OR anxiety disorder OR depression OR depressive disorder OR mood disorder OR schizophrenia OR psychosis OR psychotic OR phobia OR PTSD OR post-traumatic stress disorder OR substance use disorder OR substance abuse OR addiction OR alcohol use disorder OR ADHD OR attention deficit hyperactive disorder OR autism OR anorexia OR bulimia OR eating disorder OR binge eating OR obsessive-compulsive disorder OR dementia OR cognitive impairment OR psychiatric OR mental illness OR mental health)) |

^Note.^ A search in Google Scholar with the same keywords enabled in the finding of an additional k=1 study.
