# Supplementary material for: Evidence on Virtual Reality–Based Therapies for Psychiatric Disorders: Meta-Review of Meta-Analyses
Source: J Med Internet Res. 2020 Aug 19;22(8):e20889. doi: 10.2196/20889 (PMC7468638; doi:10.2196/20889)
Supplement: Multimedia Appendix 2 [file jmir_v22i8e20889_app2.docx]

**Supplementary Online Content**

Dellazizzo, L., Potvin, S., Luigi, M. & Dumais A. Evidence on virtual reality-based therapies for psychiatric disorders: Meta-review of meta-analyses

**Multimedia Appendix 2.** Details of the retrieved studies included in the meta-review.

This supplementary material has been provided by the authors to give readers additional information about their work.

**Multimedia Appendix 2. Details of the retrieved studies included in the meta-review.**

1. **Anxiety disorders**

| **Author, Year** | **Number of studies (Total sample size)** | **Control group (Mixed, Inactive or Active controls)** | **Time-point**  **(Post-treatment or Follow-up)** | **(i) Designs of studies included**  **(ii) Confounding/Moderators**  **(iii) Heterogeneity**  **(iv) Publication bias** | **Effect size and Confidence interval (CI)** | **Quality of evidence** |
| --- | --- | --- | --- | --- | --- | --- |
| **Acrophobia** | | | | | | |
| Parson et al. [29] | 4 (54) | Mixed:  Control including none, in vivo, waitlist | Post | (i) Mixed  (ii) Not reported  (iii) After finding evidence for the presence of heterogeneity in study outcomes, subsequent pooled analyses used random-effects estimating methods  (iv) Not reported | Cohen's d = 0.93  (within-group effect)  CI = 0.44-1.43 | Low |
| **Arachnophobia** | | | | | | |
| Parson et al. [29] | 4 (59) | Mixed:  Control including none, in vivo, waitlist | Post | (i) Mixed  (ii) Not reported  (iii) After finding evidence for the presence of heterogeneity in study outcomes, subsequent pooled analyses used random-effects estimating methods  (iv) Not reported | Cohen's d = 0.92  (within-group effect)  CI = 0.25-1.59 | Low |
| Opris et al. [28] | 2 (63) | Active:  Classical evidence-based (i.e., prolonged exposure and cognitive processing therapy) | Post | (i) RCT  (ii) Not reported  (iii) After finding evidence for the presence of heterogeneity in study outcomes, subsequent pooled analyses used random-effects estimating methods  (iv) Not reported | Cohen's d = -0.12  CI = -0.31-0.06 | Low-to-moderate |
| Opris et al. [28] | 2 (63) | Active:  Classical evidence-based (i.e., prolonged exposure and cognitive processing therapy) | Post | (i) RCT  (ii) Not reported  (iii) After finding evidence for the presence of heterogeneity in study outcomes, subsequent pooled analyses used random-effects estimating methods  (iv) Not reported | Cohen's d = -0.27  (behavioral outcome)  CI = -0.66-0.10 | Low-to-moderate |
| Opris et al. [28] | 2 (63) | Active:  Classical evidence-based (i.e., prolonged exposure and cognitive processing therapy) | Follow-up (3-6 months) | (i) RCT  (ii) Not reported  (iii) After finding evidence for the presence of heterogeneity in study outcomes, subsequent pooled analyses used random-effects estimating methods  (iv) Not reported | Cohen's d = -0.20  CI = -0.49-0.08 | Low-to-moderate |
| **Aviophobia** | | | | | | |
| Cardos et al. [30] | 16 (454) | Mixed | Post | (i) RCT  (ii) Moderators explaining efficiency:  Quality RCT & Mean age  (iii) Q=32.257; p=0.00; I^2^=53.49  (iv) Computed effect size without publication bias  Three studies with effect sizes higher than the mean, which did not change significantly the results (Adjusted effect sizes reported)  The funnel plot asymmetry suggests the presence of missing studies with effect sizes above the mean, highlighting the possibility to underestimate results of the difference | Hedges’ g = 0.592  (within-group effect)  CI = 0.327; 0.858 | Low-to-moderate |
| Cardos et al. [30] | 15 (Not reported) | Mixed | Follow-up | (i) RCT  (ii) Moderators explaining efficiency:  Number of participants & Follow-up interval  (iii) Q=44.51; p=0.00; I^2^=68.54  (iv) Computed effect size without publication bias  Three studies with effect sizes bellow the mean were estimated to significantly reduce the medium effect size of VR to a small one  The funnel plot showed some asymmetry suggesting the possibility of overestimating VR efficiency compared to control conditions at follow-up | Hedges’ g = 0.588  (within-group effect)  CI = 0.216; 0.960 | Moderate |
| Cardos et al. [30] | 4 (Not reported) | Inactive:  Control conditions-wait list and attention control | Post | (i) RCT  (ii) On account of few studies, it was not possible to perform meta-regression analysis. Moderation analysis, with categorical variable-outcome types, revealed no significant moderators  (iii) Q=9.587; p=0.02; I^2^=68.707  (iv) Computed effect size without publication bias  Estimated two studies with effect sizes lower than the mean, which did not change significantly the results  The funnel plot pointed out some asymmetry, suggesting the possibility of obtaining slightly overestimated results of VR efficiency vs. control conditions | Hedges’ g = 1.350  CI = 0.664; 2.037 | Low-to-moderate |
| Cardos et al. [30] | 2 (Not reported) | Inactive:  Control conditions-wait list and attention control | Follow-up | (i) RCT  (ii) On account of few studies, it was not possible to perform meta-regression analysis.  (iii) Q=0.154; p=0.69; I^2^=0.00  (iv) Computed effect size without publication bias | Hedges’ g = 0.583  CI = 0.108; 1.058 | Moderate |
| Cardos et al. [30] | 12 (Not reported) | Active:  Classical evidence-based (Cognitive behavioral therapy, bibliotherapy, cognitive therapy, relaxation, Cognitive behavioral therapy plus standard exposure (in vivo), relaxation techniques plus in imago exposure, computer aided exposure) | Post | (i) RCT  (ii) On account of few studies, it was not possible to perform meta-regression analysis.  (iii) Q=6.880; p=0.80; I^2^=0.00  (iv) Computed effect size without publication bias  Estimated no study with effects higher or lower than the mean, which could modify the results  Results are not affected by publication bias | Hedges’ g = 0.353  CI = 0.152; 0.555 | Moderate |
| Cardos et al. [30] | 13 (Not reported) | Active:  Classical evidence-based (Cognitive behavioral therapy, bibliotherapy, cognitive therapy, relaxation, Cognitive behavioral therapy plus standard exposure (in vivo), relaxation techniques plus in imago exposure, computer aided exposure) | Follow-up | (i) RCT  (ii) Moderators explaining efficiency:  Number of participants & Follow-up interval  (iii) Q=42.84; p=0.00; I^2^=71.99  (iv) Computed effect size without publication bias  Four studies with effect sizes bellow the mean were estimated to significantly reduce the effect size of VR  The funnel plot showed some asymmetry, suggesting the possibility that our results are affected by publication bias, overestimating VR efficiency compared to classical evidence-based interventions at follow-up | Hedges’ g = 0.615  CI = 0.179; 1.052 | Moderate |
| Cardos et al. [30] | 5 (Not reported) | Active:  Exposure-based (‘in vivo’ and ‘in imago’ exposure) | Post | (i) RCT  (ii) On account of few studies, it was not possible to perform meta-regression analysis.  (iii) Q=1.238; p=0.87; I^2^=0.00  (iv) Computed effect size without publication bias | Hedges’ g = 0.122  CI = 0.101; 1.292 | Moderate |
| Cardos et al. [30] | 9 (Not reported) | Active:  Exposure-based (‘in vivo’ and ‘in imago’ exposure) | Follow-up | (i) RCT  (ii) Moderators explaining efficiency:  Number of exposure sessions, outcome type & Follow-up interval  (iii) Q=31.45; p=0.00; I^2^=74.56  (iv) Computed effect size without publication bias  Estimated 3 studies with and effect size lower than the mean, which change significantly the results  The funnel plot pointed out some asymmetry, suggesting the possibility of overestimating results of VR efficiency versus exposure-based interventions at follow-up | Hedges’ g = 0.697  CI = 0.101; 1.292 | Moderate |
|  |  |  |  |  |  |  |
| **Panic disorder with or without agoraphobia** | | | | | | |
| Parson et al. [29] | 3 (54) | Mixed:  Control including none, in vivo, waitlist | Post | (i) Mixed  (ii) Not reported  (iii) Not reported  (iv) Not reported | Cohen's d = 1.79  (within-group effect)  CI = 1.52; 2.06 | Low |
| Opris et al. [28] | 2 (52) | Active:  Classical evidence-based (i.e., prolonged exposure and cognitive processing therapy) | Follow-up | (i) RCT  (ii) Not reported  (iii) Not reported  (iv) Not reported | Cohen's d = 0.18  CI = 0.10; 0.26 | Low-to-moderate |
| Fodor et al. [31] | 2 (16 in VR group) | Inactive:  Waitlist, placebo, treatment-as-usual | Post | (i) RCT  (ii) Conducted moderator analysis for whole sample of anxiety disorders: type of anxiety, type of control, publication bias  (iii) No heterogeneity  (iv) Whole study sample: Visual inspection pointed to an asymmetrical funnel for both anxiety: Egger’s regression intercept test was statistically significant for anxiety (intercept=2.03, 95% CI=0.07-3.98, p=0.04) | Hedges’ g = 1.80  CI = 1.01; 2.60 | Low-to-moderate |
| Fodor et al. [31] | 6 (124 in VR group) | Active:  e.g., Cognitive behavioral therapy, In vivo exposure | Post | (i) RCT  (ii) Conducted moderator analysis for whole sample of anxiety disorders: type of anxiety, type of control, publication bias  (iii) No heterogeneity  (iv) Whole study sample: Visual inspection pointed to an asymmetrical funnel for both anxiety: Egger’s regression intercept test was statistically significant for anxiety (intercept=2.03, 95% CI=0.07-3.98, p=0.04) | Hedges’ g = -0.05  CI = -0.32; 0.21 | Moderate |
| **Social anxiety** | | | | | | |
| Kampmann et al. [32] | 3 (216) | Mixed | Post | (i) RCT  (ii) Not reported  (iii) Not reported  (iv) Publication bias could not be examined since only three studies were included in the main analyses for VR | Hedges’ g = 1.09  (within-group effect)  CI = 0.80; 1.39 | Low-to-moderate |
| Kampmann et al. [32] | 2 (157) | Mixed | Follow-up (less than 5 months) | (i) RCT  (ii) Not reported  (iii) Not reported  (iv) Publication bias could not be examined | Hedges’ g = 0.93  (within-group effect)  CI = 0.46; 1.39 | Low-to-moderate |
| Kampmann et al. [32] | 2 (156) | Mixed | Follow-up (over 5 months) | (i) RCT  (ii) Not reported  (iii) Not reported  (iv) Publication bias could not be examined | Hedges’ g = 1.20  (within-group effect)  CI = 0.86; 1.54 | Low-to-moderate |
| Kampmann et al. [32] | 2 (157) | Active:  In vivo exposure | Follow-up (less than 5 months) | (i) RCT  (ii) Not reported  (iii) Not reported  (iv) Publication bias could not be examined | Hedges’ g = -0.64  CI = -1.68; 0.40 | Low-to-moderate |
| Kampmann et al. [32] | 2 (156) | Active:  In vivo exposure | Follow-up (over 5 months) | (i) RCT  (ii) Not reported  (iii) Not reported  (iv) Publication bias could not be examined | Hedges’ g = -0.01  CI = -0.39; 0.36 | Low-to-moderate |
| Carl et al. [33] | 7 (236) | Inactive:  Psychological placebo (i.e., attention control) or waitlist conditions | Post | (i) RCT  (ii) Conducted moderator analysis for whole sample of anxiety disorders: type of anxiety, type of control  (iii) I^2^=45.34 (for whole study sample)  (iv) Funnel plot asymmetry for whole study | Hedges’ g =0.97  CI = 0.62; 1.31 | Low-to-moderate |
| Chesham et al. [34] | 7 (340) | Active:  Standard treatments of in vivo or imaginal | Overall | (i) Mixed  (ii) Conducted moderator analysis for whole sample of anxiety disorders: type of anxiety, type of control  (iii) Q=10.68; p=0.099; I^2^=43.83  (iv) No asymmetry detected in the funnel plots of effect sizes | Hedges’ g =- 0.01  CI = -0.30; 0.28 | Low-to-moderate |

1. **Trauma- and stressor-related disorders (Post-traumatic stress disorder)**

| **Author, Year** | **Number of studies (Total sample size)** | **Control group (Mixed, Inactive or Active controls)** | **Time-point**  **(Post-treatment or Follow-up)** | **(i) Designs of studies included**  **(ii) Confounding/Moderators**  **(iii) Heterogeneity**  **(iv) Publication bias** | **Effect size and Confidence interval (CI)** | **Quality of evidence** |
| --- | --- | --- | --- | --- | --- | --- |
| Deng et al. [35] | 10 (309) | Mixed | Post | (i) RCT  (ii) Sub-analysis for intention-to-treat analyses and/or reported complete outcome data & Dose-response  (iii) Q=17.162; p=0.46; I^2^=47.56  (iv) No outlier studies (Eggers=0.874, p=0.425) | Hedges’ g = 0.327  (between-group effect)  CI= 0.105; 0.550 | Moderate |
| Deng et al. [35] | 5 (175) | Inactive:  Waitlist, treatment-as-usual and attention-placebo conditions | Post | (i) RCT  (ii) Not reported  (iii) Heterogeneity across the study data was not significant: Q=3.290; p=0.511; I^2^=0.511  Between-subgroup effect was significant:  Q=4.279; p=0.039  (iv) No outlier studies (Eggers=0.416, p=0.721) | Hedges’ g = 0.567  CI = 0.270; 0.863 | Low-to-moderate |
| Deng et al. [35] | 6 (239) | Active:  Cognitive behavioral therapy, exposure or other interventions | Post | (i) RCT  (ii) Not reported  (iii) Heterogeneity across the study data was not significant: Q=10.369; p=0.065; I^2^=51.781  Between-subgroup effect was significant:  Q = 4.279  p = 0.040  (iv) No outlier studies (Eggers=2.100, p=0.104) | Hedges’ g = 0 .017  CI = -0.412; 0.445 | Low-to-moderate |
| Deng et al. [35] | 9 (185) | Mixed | Follow-up (3 months) | (i) RCT  (ii) Not reported  (iii) Not reported  (iv) No outlier studies | Hedges’ g = 0.697  (between-group effect)  CI = 0.262; 1.133 | Low-to-moderate |
| Deng et al. [35] | 11 (166) | Mixed | Follow-up (6 months) | (i) RCT  (ii) Not reported  (iii) Not reported  (iv) No outlier studies | Hedges’ g = 0.848  (between-group effect)  CI = 0.324; 1.372 | Low-to-moderate |

1. **Severe mental disorders**

| **Author, Year** | **Number of studies (Total sample size)** | **Control group (Mixed, Inactive or Active controls)** | **Time-point**  **(Post-treatment or Follow-up)** | **(i) Designs of studies included**  **(ii) Confounding/Moderators**  **(iii) Heterogeneity**  **(iv) Publication bias** | **Effect size and Confidence interval (CI)** | **Quality of evidence** |
| --- | --- | --- | --- | --- | --- | --- |
| **Depressive disorder** | | | | | | |
| Kampann et al. [32] | 2 (19) | Mixed | Post | (i) RCT  (ii) Not reported  (iii) Not reported  (iv) Not reported | Hedges’ g = 0.44  (within-group effect)  CI = 0.02; 0.87 | Low-to-moderate |
| Fodor et al., [31] | 10 (Not reported) | Inactive:  Waitlist, placebo, treatment-as-usual | Post | (i) RCT  (ii) Subgroup analysis for publication year in the whole sample of the study  (iii) I^2^=71%  (iv) Whole study sample: Visual inspection pointed to an asymmetrical funnel for depression: Egger’s regression intercept test was statistically significant for depression outcomes (intercept=3.24, 95% CI=0.10 to 6.39, p=0.04) | Hedges’ g = 0.73  CI = 0.25; 1.21 | Low-to-moderate |
| Fodor et al., [31] | 13 (Not reported) | Active:  e.g., Cognitive behavioral therapy, In vivo exposure | Post | (i) RCT  (ii) Subgroup analysis for publication year in the whole sample of the study  (iii) I^2^=26%  (iv) Whole study sample: Visual inspection pointed to an asymmetrical funnel for depression: Egger’s regression intercept test was statistically significant for depression outcomes (intercept=3.24, 95% CI=0.10 to 6.39, p=0.04) | Hedges’ g = 0.004  CI = -0.20; 0.21 | Moderate |
| Fodor et al., [31] | 5 (Not reported) | Active:  e.g., Cognitive behavioral therapy, In vivo exposure | Follow-up | (i) RCT  (ii) Subgroup analysis for publication year in the whole sample of the study  (iii) I^2^=57%  (iv) Whole study sample: Visual inspection pointed to an asymmetrical funnel for depression: Egger’s regression intercept test was statistically significant for depression outcomes (intercept=3.24, 95% CI=0.10 to 6.39, p=0.04) | Hedges’ g = -0.19  CI = -0.62; 0.23 | Moderate |
| Deng et al. [35] | 7 (209) | Mixed | Post | (i) RCT  (ii) Not reported  (iii) Not reported  (iv) Not reported | Hedges’ g = 0.373  (between-group effect)  CI = 0.110; 0.637 | Low |
| **Schizophrenia spectrum** | | | | | | |
| Valimaki et al. [36] | 3 (156) | Mixed | Lost to follow up (5-12 weeks) | (i) RCT  (ii) Not reported  (iii) tau^2^=0; chi^2^=1.01; df=2 (p=0.6); I^2^=0%  (iv) There is, at the very least, a moderate risk of bias in all outcomes and therefore a risk of overestimating any positive effects of VR for people with serious mental illness | Risk difference = 0.02  (between-group effect)  CI = -0.08; 0.12 | Moderate |

1. **Neurodevelopmental disorder (Autism)**

| **Author, Year** | **Number of studies (Total sample size)** | **Control group (Mixed, Inactive or Active controls)** | **Time-point**  **(Post-treatment or Follow-up)** | **(i) Designs of studies included**  **(ii) Confounding/Moderators**  **(iii) Heterogeneity**  **(iv) Publication bias** | **Effect size and Confidence interval (CI)** | **Quality of evidence** |
| --- | --- | --- | --- | --- | --- | --- |
| Barton et al. [37] | 2 (30) | Mixed:  Any comparison group | Post | (i) Mixed  (ii) Not reported  (iii) Not reported  (iv) Whole study sample: The distribution of effect size estimates appears to be somewhat asymmetrical, with smaller effects tending to be more precisely estimated and larger effects tending to be less precise.  This visual assessment of asymmetry is consistent with robust Egger’s regression tests, t(4.9)=4.05, p=0.010. | Hedges’ g = 0.37  (between-group effect)  CI = -1.71-2.46 | Very Low |

1. **Neurocognitive disorders (Mild cognitive impairment/Dementia)**

| **Author, Year** | **Number of studies (Total sample size)** | **Control group (Mixed, Inactive or Active controls)** | **Time-point**  **(Post-treatment or Follow-up)** | **(i) Designs of studies included**  **(ii) Confounding/Moderators**  **(iii) Heterogeneity**  **(iv) Publication bias** | **Effect size and Confidence interval (CI)** | **Quality of evidence** |
| --- | --- | --- | --- | --- | --- | --- |
| Kim et al. [38] | 11 (271) | Mixed | Post | (i) Mixed  (ii) Sub-analysis by:  Patient type, Setting, Control group versus no control group, Randomized allocation, VR task, Intervention outcome  (iii) Significant Q statistics (p<0.01) were identified as heterogeneous, Q=21.572  (iv) Because they needed a large number of studies to nullify the effect, they say publication bias was not a concern | Cohen's d=0.29  (mixed impairments; within-group effect)  CI = 0.16; 0.42 | Low-to-moderate |
| Kim et al. [38] | Not reported (Not reported) | Mixed | Post | (i) Mixed  (ii) Not reported  (iii) Not reported  (iv) Not reported | Cohen's d = 0.41  (physical fitness; within-group effect)  CI = 0.16; 0.65 | Low |
| Kim et al. [38] | Not reported (Not reported) | Mixed | Post | (i) Mixed  (ii) Not reported  (iii) Not reported  (iv) Not reported | Cohen's d = 0.42  (cognition; within-group effect)  CI = 0.24; 0.60 | Low-to-moderate |
| Kim et al. [38] | Not reported (Not reported) | Mixed | Post | (i) Mixed  (ii) Not reported  (iii) Not reported  (iv) Not reported | Cohen's d = 0.14  (emotion; within-group effect)  CI = -0.07; 0.36 | Low-to-moderate |
| Kim et al. [38] | Not reported (Not reported) | Mixed | Post | (i) Mixed  (ii) Not reported  (iii) Not reported  (iv) Not reported | Cohen's d = 0.07  (execution; within-group effect)  CI = -0.34; 0.49 | Low |
